# Dr. Adams *on Muscæ Volitantes*

**Published:** 1818-02

**Authors:** 


*For the London Medical and Physical Journal.*

### Dr. Adams *on Muscæ Volitantes.*

THE late Mr. Ware, in his ingenious paper on Muscæ Volitantes, contained in the fifth volume of the Medico-Chirurgical Transactions, imputed this appearance to some pressure on the optic nerve. He gives three well-recorded cases. Though he considers the disease generally as the effect of some irregularity in the nervous system, yet his third case can scarcely be imputed to this cause.* His paper closes with an ingenious communication from a medical gentleman, giving an account of his own case, which, as Mr. Ware remarks, appears to have been the "*suffusio scintillans*" of Sauvages. Whether any medical writer has described muscæ volitantes by his own sensation, I do not pretend to determine; but, as nothing of the kind has occurred in my reading, a record of what I recently observed in my own eye, will, I trust, not be thought obtrusive on the pages of the Journal.

About a month ago, when in perfect health, on rising in the morning, I perceived the appearance of a large black fly immediately before my left eye.—It moved and was alternately still, but never out of sight, so that in a short time there could be no doubt that the image, whatever it might be, was attached to the eye. At first, it seemed really a small fly entangled in the lashes, and, under this impression, I made haste to wash the eye; but to no purpose. The image was constantly before me, moving as my eye moved, and, when that was steady, seeming gradually to descend, but only to a certain point. In all other respects, vision was perfect, and as this shadow, if I may so call it, was not in the centre, the irksomeness of its intrusion was the chief inconvenience experienced.

After being teazed with it for the whole day, on the following morning it was first perceived whilst my eye was closed, and when the light of the room was by no means strong. I could now no longer keep from asking the opinion of my friend, Mr. Stevenson; who, on applying a large magnifier, perceived something as if hanging from the upper part of the iris; but, as the rest of the eye appeared clear, and as my sight was not in other respects impaired, he encouraged me to expect the gradual absorption of the substance, whatever it might be.

On the day following, the spectrum was somewhat lengthened, but not so broad from above downwards. In two days after, the end nearest the nose was produced into two long streaks, like the long legs of some gnat, but still perfectly black and opake. In a few days more these legs were separated, and the body seemed broken in one or two places. After this, it gave the appearance of two or three smaller flies, and, in about three weeks, nothing was left but

---

* This paper is slightly noticed in our 33d volume, p. 411, but, having devoted much of this Number to ophthalmic subjects, we have transcribed Mr. W.'s cases in our Collectanea.

several minute dots, most of them black and opake, but some nearly transparent.

Encouraged by this amendment, and by the consolatory language of Mr. Stevenson, I had now leisure and spirits to reason on the subject. The most probable cause of the whole seemed to me the detachment of a portion of that substance called the *pigmentum nigrum*, which had probably fallen on the anterior part of the lens. I can hardly conceive it was floating loose in the aqueous humour, as its situation was certainly fixed, and its apparent motion depended only on the accidental intrusion between the eye and objects obliquely situated before it, as the whole globe of the eye was moved. The gradual change in the appearance of the spectrum—the manner in which it seemed to break, reminded me perpetually of the slow disappearance of a part of the capsule after the operation of couching. In this I was confirmed by Mr. Stevenson, who, after minute examination, detected a gradual lessening of the substance.

It will be presumed, that the eye was unattended with inflammation, and, I have before observed, my health and spirits were good during the whole period from the time I was relieved of my first apprehension.

I shall conclude with a few remarks on the three cases related by Mr. Ware. Of the first, it is worth noticing, that the patient was not conscious that the complaint was confined to one eye, till, by Mr. Ware's assistance, the fact was ascertained ;—a proof, among many others, of the uncertainty of the accounts we receive from our patients, and how necessary it is to extend our own examination to every thing which may afford information. The second case was given by a person apparently more careful in his descriptions ; but the account in one part of the history does not well accord with the idea of an insect under the upper right eye-lid. This is the only case in which, we are informed, the *spectra* were seen almost as strongly when the eye-lids were open as when closed. In the third, I shall only remark, that the moats assumed the appearance of fixed figures. When the eyes were shut, the appearance was not exactly similar.

It is seldom that any two cases are exactly alike. My own partook of some of the particulars of each of the above three, but was not completely the same as either. This may be imputed, partly to different stages of the complaint described to Mr. Ware, and, perhaps, to some difference in the colour of the pigmentum, which is not exactly the same in all persons.

Mr. Ware, and most others, are of opinion, that this affection of the eye must be the effect of some pressure on a part of the optic nerve, not immediately within the axis of vision, or some nervous irritation, and urges, that the outlines of the spectrum are usually undefined or obscure. Having felt no pain, and none of those sensations usually called nervous, I cannot consider my own case as arising from either of those causes. The outlines of the spectrum were perfectly defined, and could not be called obscure, excepting that every attempt to bring the object within the direct axis of vision was abortive. I could, however, so far direct my whole attention to it as to survey its figure with scrupulous exactness.

I am not aware, that any writers, whether medical or purely philosophical, have admitted a substance opposed between the nerve and the cornea, so as to intercept rays passing in any direction. I shall, therefore, be anxious for the confirmation of any medical gentleman who may experience a similar inconvenience, or may have a fair opportunity of tracing its progress through all its stages, in an intelligent and inquisitive patient.

*Hatton-garden; Jan.* 1818.

---

